# Next-Generation Sequencing Analysis of *GBA1*: The Challenge of Detecting Complex Recombinant Alleles

**DOI:** 10.3389/fgene.2021.684067

**Published:** 2021-06-21

**Authors:** Elizabeth G. Woo, Nahid Tayebi, Ellen Sidransky

**Affiliations:** Medical Genetics Branch, National Human Genome Research Institute, National Institutes of Health, Bethesda, MD, United States

**Keywords:** next-generation sequencing, glucocerebrosidase, Gaucher disease, *GBA1*, pseudogene, genotyping

## Introduction

Gaucher disease (GD) is a rare autosomal recessive lysosomal storage disorder caused by pathologic variants in *GBA1*, the gene encoding the enzyme glucocerebrosidase. Deficiency of glucocerebrosidase leads to the accumulation of the substrates glucocerebroside and glucosylsphingosine in macrophages and neuronal cells. Variants in *GBA1* are a significant genetic risk factor for Define- Parkinson disease (PD) (Sidransky et al., 2009). Both patients with GD and heterozygous carriers are at an increased risk of developing PD, although the exact mechanism of this association is not fully understood (Avenali et al., 2020). An estimated 5–20% of patients with PD carry a *GBA1* mutation, although the frequency varies between populations (Gan-Or et al., 2015). The association of *GBA1* variants with this common complex disorder has led to an increasing interest in analyzing *GBA1* in larger cohorts using next-generation sequencing (NGS) methods (Nalls et al., 2014; Gorostidi et al., 2016; Blauwendraat et al., 2018; Stoker et al., 2020). Challenges arise in using NGS for *GBA1* analysis, however, due to the downstream, highly homologous pseudogene.

**Figure 1:**
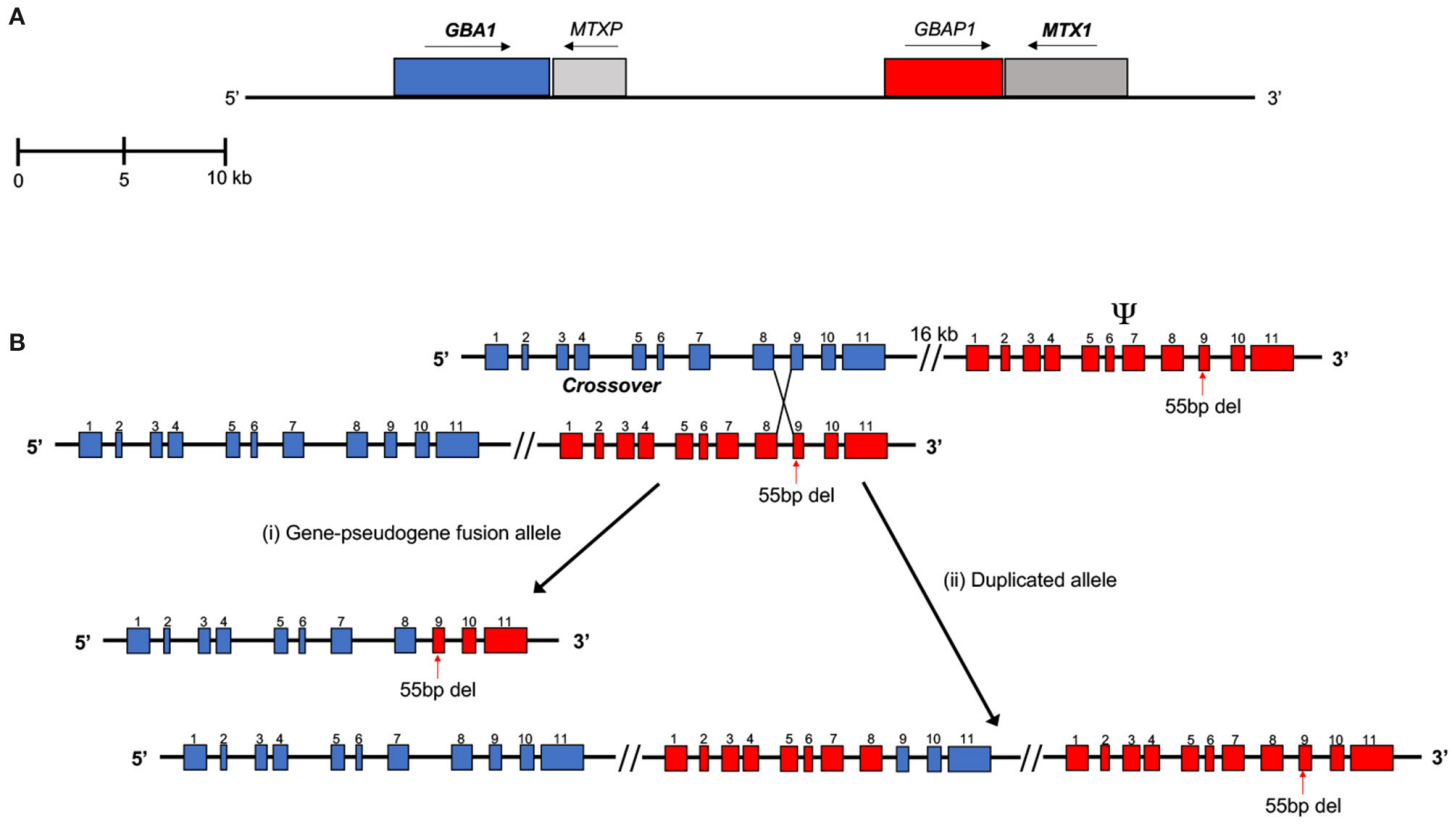
**(A)** Scaled representation of *GBA1, MTX1*, and their highly homologous pseudogenes. *MTXP* lies in the 16 kb region between *GBA1* and *GBAP1*. *MTX1* and *MTXP* are transcribed in the direction opposite to that of *GBA1* and *GBAP1*. **(B)** Example of a reciprocal crossover event, resulting in a (i) gene-pseudogene fusion allele and a (ii) duplicated allele. These recombinant alleles are difficult to detect using next-generation sequencing methods. Sequence reads containing the pseudogene-derived segment (red) of the recombinant alleles may align to the pseudogene instead. This can lead to false negatives and reduce the read depth of *GBA1*. In this example, reads containing the 55 bp deletion from the recombinant alleles may align preferentially to the pseudogene (Adapted in part from Tayebi et al., 2003).

*GBA1* is located in a gene-rich region on chromosome 1q21 that encompasses seven genes and two pseudogenes within an 85-kb region (Winfield et al., 1997). *GBA1* contains 11 exons and 10 introns over a length of 7.6 kb. Located 16 kb downstream is the shorter (5.7 kb), non-processed pseudogene, *GBAP1* (Horowitz et al., 1989). In the coding regions, *GBAP1* is 96% homologous to the functional gene, increasing to around 98% in the region between intron 8 and the 3′ UTR. However, *GBAP1* lacks a 55-bp segment in exon 9, which is the major exonic difference between the two sequences (Walley and Harris, 1993; Beutler et al., 1995; Tayebi et al., 1996b). *GBA1* has several *Alu* intronic insertions that are not present in *GBAP1*, indicating that evolutionarily, the gene duplication occurred prior to the integration of the *Alu* sequences (Horowitz et al., 1989). The closest downstream gene, located just beyond *GBAP1* is metaxin (*MTX1*), encoding for part of the mitochondrial outer membrane import complex protein 1, which is transcribed convergently to *GBA1* (Long et al., 1996). *MTX1* also has a pseudogene located in the 16-kb region between *GBA1* and *GBAP1* (Figure 1A). Several hundred mutations have been identified in *GBA1* including missense mutations, deletions, insertions, splice site mutations, and complex recombinant alleles (Hruska et al., 2008).

The presence of these pseudogenes in this region increases the risk for recombination and the generation of complex alleles, particularly due to the close physical proximity and high homology between *GBA1* and *GBAP1* (Chen et al., 1997). Recombinant alleles can arise from both reciprocal and non-reciprocal recombination, resulting in gene fusions, duplications, and gene conversion alleles (Cormand et al., 2000; Tayebi et al., 2003). These genes can serve as a model to demonstrate the effect of unequal pairing and crossover resulting in recombinant alleles in the genome. Identifying complex alleles poses a significant challenge when relying on NGS analysis.

## *GBA1* Genotyping by Conventional Means

Previously, PCR-based screening techniques were often used targeting specific panels of 7–10 common *GBA1* pathologic variants (Park et al., 2002). These variants were predicted to account for over 90% of mutant alleles among Ashkenazi Jews, a population with a carrier rate of ~1 in 14 (Do et al., 2019). However, relying on such panels can miss other mutant alleles, as demonstrated by a 2019 study that fully sequenced *GBA1* in an Ashkenazi Jewish Parkinson disease patient cohort (Ruskey et al., 2019). Sanger sequencing substantially increased variant detection, even in this group, particularly for the common E326K (p.E365K) variant, known to be present in ~1% of the general population (Lau et al., 1999; Park et al., 2002). This highlights the benefit of sequencing the full *GBA1* gene to detect variants that could be missed using targeted genotyping of selected pathogenic variants. When sequencing *GBA1*, specific primers are required to ensure that functional *GBA1* is being amplified and sequenced rather than the pseudogene (Dandana et al., 2016). For example, primers targeted to the 55-bp pseudogene gap in exon 9 can erroneously misreport patients heteroallelic for a recombination in that region as homozygosity for the second allele (Tayebi et al., 1996b).

## Next-Generation Sequencing of *GBA1*

Next-generation sequencing is able to generate vast amounts of sequence data and has been applied to identify variants in *GBA1*. The use of whole-exome and whole-genome sequencing has increased the discovery rate of these variants. For example, a recent large-scale NGS strategy identified 18 novel *GBA1* variants, as well as a rarely reported complex allele likely to be a Dutch founder allele (Zampieri et al., 2017; den Heijer et al., 2020). One Gaucher diagnostics laboratory relying on NGS run by Centogene (Rostock, Germany) provides an on-line list of their identified *GBA1* variants (CentoLSD™), which now includes over 600 different *GBA1* variants with differing predicted clinical significance (https://www.centogene.com/centolsd.html). Many NGS protocols have utilized short-read sequencing, which can provide data at a relatively low cost and high accuracy but may be less reliable in cases where there are structural variants and repetitive or highly homologous regions (Mandelker et al., 2016; Mantere et al., 2019). If the functional gene is not specifically amplified during library preparation, reads generated by the pseudogene could align with the functional gene, leading to false-positive results, while reads belonging to the functional gene might align to the pseudogene, reducing read depth and mapping quality (Claes and De Leeneer, 2014). Long range-PCR (LR-PCR) can help to selectively amplify the functional gene (Tayebi et al., 1996a; Jeong et al., 2011). Even after selectively amplifying the functional gene, challenges still remain in the alignment phase, particularly when recombinant alleles contain regions identical to the pseudogene. In an Illumina short-read NGS method for sequencing *GBA1*, Zampieri et al. initially aligned reads to the whole genome, but missed almost all recombinant alleles, which had aligned preferentially to *GBAP1* (Zampieri et al., 2017). After aligning to *GBA1* rather than the whole genome, they were able to identify recombinant variants that had initially been false negatives, except for the 55-bp deletion mutation. Alignment against the whole genome did not significantly affect the mapping quality and coverage of missense mutations unrelated to recombination. Care should therefore be taken not only in the library preparation, but also in data analysis steps of NGS pipelines to sequence *GBA1*.

The introduction of long-read sequencing technology in recent years addressed some limitations of short-read sequencing (Goodwin et al., 2016). The ability to read longer sequences can help to differentiate highly homologous regions. One such technology is the Oxford Nanopore system, which analyzes a single DNA molecule as it passes through a pore (Logsdon et al., 2020). Resulting disruptions to the current are analyzed in real-time to determine the sequence. This strategy has been applied for the detection of *GBA1* variants using the Oxford Nanopore MinION (Leija-Salazar et al., 2019). The authors were able to detect the 55-bp exonic deletion, but not the more common recombinant allele Rec*NciI*. Another study aimed to expand upon this protocol by refining and applying it to the discovery of *GBA1* variants in a PD longitudinal cohort from New Zealand (Graham et al., 2020). They validated the protocol of Leija-Salazar et al. for genotyping *GBA1* and updated the software pipeline, improving accuracy and reducing the computational workload, but were unable to detect any recombinant gene conversion alleles or deletions of >50 bp.

## Challenges in Detecting Recombinant Alleles

Accurate and comprehensive NGS analysis of *GBA1* is complicated by complex gene-pseudogene rearrangements and recombination. Recombinant alleles make up a significant proportion of *GBA1* mutations and sites of recombination events have been identified between intron 2 to exon 11 (Hruska et al., 2008). With the high exonic homology between *GBA1* and its pseudogene, there is an increased likelihood of both reciprocal and non-reciprocal recombination (Tayebi et al., 2003). These can result in alleles with multiple exonic point mismatches, such as in Rec*NciI*. In this allele, the site of crossover of chromosomal mispairing between gene and pseudogene occurs within intron 9 and continues to the 3'-UTR of the gene, introducing three exon 10 nucleotide mismatches (Latham et al., 1990). Another frequently described reciprocal recombinant allele, *RecTL*, covers the pseudogene sequence from intron 8 or the beginning of exon 9 [Figure 1B(i)] (Zimran and Horowitz, 1994). Reciprocal crossover can also result in a partial duplication of the gene and pseudogene sequence [Figure 1B(ii)]. The non-reciprocal exchange of homologous sequence, also known as gene conversion, may also occur. One example is an allele that includes only the 55-bp deletion in exon 9. This is a small, converted gene sequence that resembles the pseudogene. Some polymorphic sequences in *GBAP1* (Martinez-Arias et al., 2001) also been observed. Mostly, homologous recombination events occur in regions of high sequence homology between the gene and pseudogene, such as in introns 8 and 9. Several instances of recombination have occurred as a result of crossover between *MTX* and its pseudogene as well (Tayebi et al., 2003). Quantitative real-time PCR and Southern blot analysis have been used to identify fusions and duplications in patients with recombinant *GBA1* alleles (Velayati et al., 2011).

The complexity of *GBA1* gene rearrangements cannot be sufficiently captured using most current NGS methods. Several recent studies using NGS technology without Sanger sequencing validation have not reported the presence of recombinant alleles, including a recent study performed on more than 3,000 PD cases (den Heijer et al., 2020). Thus, it is likely that the results presented may be an underestimation (Zampieri et al., 2017).

## Non-Conventional Inheritance

Gaucher disease is an autosomal recessive disorder that usually results from the inheritance of a mutant allele from each parent. Increasing evidence suggests this may not always be the case, and that there are important exceptions to the traditional Mendelian pattern of inheritance, such as new mutations or uniparental disomy (Nakka et al., 2019). There have been several reported cases of unrelated patients with type 2 GD with *de novo* or germline mutations on the maternal allele (Saranjam et al., 2013; Hagege et al., 2017). Uniparental disomy of chromosome 1 was reported in a proband with concurrent type 3 GD and Charcot-Marie-Tooth disease, who was homozygous for both L444P (p.L483P) in *GBA1* and S78L in *MPZ* (Benko et al., 2008). There have also been cases of patients with all types of GD carrying more than one *GBA1* mutation on the same allele (Hassan et al., 2018). For example, a common mutant allele in Greece and the Balkans includes both H255Q (p.H255Q) and D409H (p.D448H) together in cis (Vithayathil et al., 2009). These examples of non-conventional inheritance underscore the importance of careful and comprehensive examination of entire coding regions for accurate genotyping. This has important implications for diagnostics and establishing genotype-phenotype correlations. Current NGS technology, however, does not always accurately identify all nucleotide changes and recombinant alleles. As a result, NGS is currently limited in its clinical diagnostic capacity for comprehensive *GBA1* screening, and for now, Sanger sequencing should be used for the most accurate genotyping.

## Discussion

Next-generation sequencing allows for unbiased simultaneous analysis of many genes. In addition, it makes it more feasible to analyze specific genes in larger cohorts for the study of common diseases like Parkinson disease. With the inclusion of *GBA1* in many Parkinson disease NGS analyses, it is important to consider the effects of the nearby homologous pseudogene. Recombinant alleles in *GBA1* have been identified in patients with GD and with PD that might be missed when relying on NGS analysis alone without Sanger sequencing validation.

Challenges exist in short-read NGS methods for sequencing highly homologous regions. The shorter reads cannot be mapped uniquely to the reference genome, especially in cases where there are recombinant alleles aligning to the homologous region. A computational custom scaffold-based approach was recently introduced to improve the detection and phasing of targeted complex variants using short reads (Zeng et al., 2020). This method was able to detect a 55-bp deletion in *GBA1* confirmed by Sanger sequencing. In addition, there is evidence to suggest that the choice of polymerase used could be a factor in the accuracy of NGS variant identification. A recent study performed a large-scale screening of *GBA1* based on an NGS protocol and found a significant number of false negatives due to a polymerase-dependent allelic imbalance (den Heijer et al., 2021). After performing a structured assessment of varied PCR conditions, they found that changing the polymerase used led to the resolution of these false negatives. Allele frequency was unaffected by a change in the other conditions. This raises the possibility that current estimates of variant frequency in populations could be underestimates, due to polymerase-dependent false negatives as well.

Long-read NGS has many advantages, one of which is an improved ability to discriminate functional genes such as *GBA1* from their pseudogenes. An additional advantage is the ability to phase mutations and assign haplotypes. It can also detect intronic SNPs. There are still some limitations, including high costs, low throughput, and high per-base error rates. Long-read NGS also has a limited ability to accurately resolve homopolymers and to detect small insertions and deletions. Importantly, it is still unable to consistently detect recombinant alleles.

The most accurate method for detecting all *GBA1* variants and recombination remains Sanger sequencing. Without validation by Sanger sequencing, the frequencies of *GBA1* variants based on NGS analysis may be underestimated, particularly for complex recombinant alleles. Real-time PCR can also be used to identify recombinant alleles (Velayati et al., 2011). NGS pipelines should be carefully designed in order to capture variants should from the functional gene rather than the pseudogene and attempt to include complex alleles. While long-read sequencing shows promise for increased accuracy of *GBA1* NGS analysis, it is still currently limited in its capacity to recognize recombinant alleles. The shortcomings identified will likely also be pertinent for the analysis of other genes with highly homologous pseudogenes. As sequencing technology continues to rapidly progress, we will likely continue to see improved detection of *GBA1* variants. This has exciting potential for clinical diagnostics and studies of large patient cohorts.

## Author Contributions

EW: drafted the manuscript and figure. NT: organized the topic, reviewed the literature, and edited the manuscript. ES: conceived of the topic and edited the manuscript. All authors contributed to the article and approved the submitted version.

## Conflict of Interest

The authors declare that the research was conducted in the absence of any commercial or financial relationships that could be construed as a potential conflict of interest.

## References

[B1] Avenali M. Blandini F. Cerri S. (2020). Glucocerebrosidase defects as a major risk factor for Parkinson's Disease. Front. Aging Neurosci. 12:97. 10.3389/fnagi.2020.00097 32372943 PMC7186450

[B2] Benko W. S. Hruska K. S. Nagan N. Goker-Alpan O. Hart P. S. Schiffmann R.. (2008). Uniparental disomy of chromosome 1 causing concurrent Charcot-Marie-Tooth and Gaucher disease Type 3. Neurology 70, 976–978. 10.1212/01.wnl.0000305963.37449.32 18347322 PMC4617237

[B3] Beutler E. Gelbart T. Demina A. Zimran A. LeCoutre P. (1995). Five new Gaucher disease mutations. Blood Cells Mol. Dis. 21, 20–24. 10.1006/bcmd.1995.0004 7655857

[B4] Blauwendraat C. Bras J. M. Nalls M. A. Lewis P. A. Hernandez D. G. Singleton A. B.. (2018). Coding variation in GBA explains the majority of the SYT11-GBA Parkinson's disease GWAS locus. Mov. Disord. 33, 1821–1823. 10.1002/mds.103 30302829 PMC6379910

[B5] Chen K. S. Manian P. Koeuth T. Potocki L. Zhao Q. Chinault A. C.. (1997). Homologous recombination of a flanking repeat gene cluster is a mechanism for a common contiguous gene deletion syndrome. Nat. Genet. 17, 154–163. 10.1038/ng1097-154 9326934

[B6] Claes K. B. De Leeneer K. (2014). Dealing with pseudogenes in molecular diagnostics in the next-generation sequencing era. Methods Mol. Biol. 1167, 303–315. 10.1007/978-1-4939-0835-6_21 24823787

[B7] Cormand B. Diaz A. Grinberg D. Chabas A. Vilageliu L. (2000). A new gene-pseudogene fusion allele due to a recombination in intron 2 of the glucocerebrosidase gene causes Gaucher disease. Blood Cells Mol. Dis. 26, 409–416. 10.1006/bcmd.2000.0317 11112377

[B8] Dandana A. Ben Khelifa S. Chahed H. Miled A. Ferchichi S. (2016). Gaucher disease: clinical. biological and therapeutic aspects. Pathobiology 83, 13–23. 10.1159/000440865 26588331

[B9] den Heijer J. M. Cullen V. C. Quadri M. Schmitz A. Hilt D. C. Lansbury P.. (2020). A large-scale full GBA1 gene screening in Parkinson's disease in the Netherlands. Mov. Disord. 35, 1667–1674. 10.1002/mds.28112 32618053 PMC7540512

[B10] den Heijer J. M. Schmitz A. Lansbury P. Cullen V. C. Hilt D. C. Bonifati V.. (2021). False negatives in GBA1 sequencing due to polymerase dependent allelic imbalance. Sci. Rep. 11:161. 10.1038/s41598-020-80564-y 33420335 PMC7794395

[B11] Do J. McKinney C. Sharma P. Sidransky E. (2019). Glucocerebrosidase and its relevance to Parkinson disease. Mol. Neurodegener. 14:36. 10.1186/s13024-019-0336-2 31464647 PMC6716912

[B12] Gan-Or Z. Amshalom I. Kilarski L. L. Bar-Shira A. Gana-Weisz M. Mirelman A.. (2015). Differential effects of severe vs mild GBA mutations on Parkinson disease. Neurology 84, 880–887. 10.1212/WNL.0000000000001315 25653295 PMC4351661

[B13] Goodwin S. McPherson J. D. McCombie W. R. (2016). Coming of age: ten years of next-generation sequencing technologies. Nat. Rev. Genet. 17, 333–351. 10.1038/nrg.2016.49 27184599 PMC10373632

[B14] Gorostidi A. Marti-Masso J. F. Bergareche A. Rodriguez-Oroz M. C. Lopez de Munain A. Ruiz-Martinez J. (2016). Genetic mutation analysis of Parkinson's disease patients using multigene next-generation sequencing panels. Mol. Diagn. Ther. 20, 481–491. 10.1007/s40291-016-0216-1 27294386

[B15] Graham O. E. E. Pitcher T. L. Liau Y. Miller A. L. Dalrymple-Alford J. C. Anderson T. J.. (2020). Nanopore sequencing of the glucocerebrosidase (GBA) gene in a New Zealand Parkinson's disease cohort. Parkinsonism Relat. Disord. 70, 36–41. 10.1016/j.parkreldis.2019.11.022 31809948

[B16] Hagege E. Grey R. J. Lopez G. Roshan Lal T. Sidransky E. Tayebi N. (2017). Type 2 Gaucher disease in an infant despite a normal maternal glucocerebrosidase gene. Am. J. Med. Genet. A. 173, 3211–3215. 10.1002/ajmg.a.38487 29091352 PMC5787391

[B17] Hassan S. Lopez G. Stubblefield B. K. Tayebi N. Sidransky E. (2018). Alleles with more than one mutation can complicate genotype/phenotype studies in Mendelian disorders: lessons from Gaucher disease. Mol. Genet. Metab. 125, 1–3. 10.1016/j.ymgme.2018.06.013 29980418 PMC6178817

[B18] Horowitz M. Wilder S. Horowitz Z. Reiner O. Gelbart T. Beutler E. (1989). The human glucocerebrosidase gene and pseudogene: structure and evolution. Genomics 4, 87–96. 10.1016/0888-7543(89)90319-4 2914709

[B19] Hruska K. S. LaMarca M. E. Scott C. R. Sidransky E. (2008). Gaucher disease: mutation and polymorphism spectrum in the glucocerebrosidase gene (GBA). Hum. Mutat. 29, 567–583. 10.1002/humu.20676 18338393

[B20] Jeong S. Y. Kim S. J. Yang J. A. Hong J. H. Lee S. J. Kim H. J. (2011). Identification of a novel recombinant mutation in Korean patients with Gaucher disease using a long-range PCR approach. J. Hum. Genet. 56, 469–471. 10.1038/jhg.2011.37 21490608

[B21] Latham T. Grabowski G. A. Theophilus B. D. Smith F. I. (1990). Complex alleles of the acid beta-glucosidase gene in Gaucher disease. Am. J. Hum. Genet. 47, 79–86. 2349952 PMC1683763

[B22] Lau E. K. Tayebi N. Ingraham L. J. Winfield S. L. Koprivica V. Stone D. L.. (1999). Two novel polymorphic sequences in the glucocerebrosidase gene region enhance mutational screening and founder effect studies of patients with Gaucher disease. Hum. Genet. 104, 293–300. 10.1007/s004390050957 10369158

[B23] Leija-Salazar M. Sedlazeck F. J. Toffoli M. Mullin S. Mokretar K. Athanasopoulou M.. (2019). Evaluation of the detection of GBA missense mutations and other variants using the Oxford Nanopore MinION. Mol. Genet. Genomic Med. 7:e564. 10.1002/mgg3.564 30637984 PMC6418358

[B24] Logsdon G. A. Vollger M. R. Eichler E. E. (2020). Long-read human genome sequencing and its applications. Nat. Rev. Genet. 21, 597–614. 10.1038/s41576-020-0236-x 32504078 PMC7877196

[B25] Long G. L. Winfield S. Adolph K. W. Ginns E. I. Bornstein P. (1996). Structure and organization of the human metaxin gene (MTX) and pseudogene. Genomics 33, 177–184. 10.1006/geno.1996.0181 8660965

[B26] Mandelker D. Schmidt R. J. Ankala A. McDonald Gibson K. Bowser M. Sharma H.. (2016). Navigating highly homologous genes in a molecular diagnostic setting: a resource for clinical next-generation sequencing. Genet. Med. 18, 1282–1289. 10.1038/gim.2016.58 27228465

[B27] Mantere T. Kersten S. Hoischen A. (2019). Long-read sequencing emerging in medical genetics. Front. Genet. 10:426. 10.3389/fgene.2019.00426 31134132 PMC6514244

[B28] Martinez-Arias R. Comas D. Mateu E. Bertranpetit J. (2001). Glucocerebrosidase pseudogene variation and Gaucher disease: recognizing pseudogene tracts in GBA alleles. Hum. Mutat. 17, 191–198. 10.1002/humu.4 11241841

[B29] Nakka P. Pattillo Smith S. O'Donnell-Luria A. H. McManus K. F. Team andMe Research Mountain, J. L.. (2019). Characterization of prevalence and health consequences of uniparental disomy in four million individuals from the general population. Am. J. Hum. Genet. 105, 921–932. 10.1101/540955 31607426 PMC6848996

[B30] Nalls M. A. Pankratz N. Lill C. M. Do C. B. Hernandez D. G. Saad M.. (2014). Large-scale meta-analysis of genome-wide association data identifies six new risk loci for Parkinson's disease. Nat. Genet. 46, 989–993. 10.1038/ng.3043 25064009 PMC4146673

[B31] Park J. K. Tayebi N. Stubblefield B. K. LaMarca M. E. MacKenzie J. J. Stone D. L.. (2002). The E326K mutation and Gaucher disease: mutation or polymorphism? Clin. Genet. 61, 32–34. 10.1034/j.1399-0004.2002.610106.x 11903352

[B32] Ruskey J. A. Greenbaum L. Ronciere L. Alam A. Spiegelman D. Liong C.. (2019). Increased yield of full GBA sequencing in Ashkenazi Jews with Parkinson's disease. Eur. J. Med. Genet. 62, 65–69. 10.1016/j.ejmg.2018.05.005 29842932 PMC6261782

[B33] Saranjam H. Chopra S. S. Levy H. Stubblefield B. K. Maniwang E. Cohen I. J.. (2013). A germline or de novo mutation in two families with Gaucher disease: implications for recessive disorders. Eur. J. Hum. Genet. 21, 115–117. 10.1038/ejhg.2012.105 22713811 PMC3522207

[B34] Sidransky E. Nalls M. A. Aasly J. O. Aharon-Peretz G. Annesi E. R. Barbosa E. R.. (2009). Multi-center analysis of glucocerebrosidase mutations in Parkinson disease. N. Engl. J. Med. 361, 1651–1661. 10.1056/NEJMoa0901281 19846850 PMC2856322

[B35] Stoker T. B. Camacho M. Winder-Rhodes S. Liu G. Scherzer C. R. Foltynie T.. (2020). Impact of GBA1 variants on long-term clinical progression and mortality in incident Parkinson's disease. J. Neurol. Neurosurg. Psychiatry 91, 695–702. 10.1136/jnnp-2020-322857 32303560 PMC7361014

[B36] Tayebi N. Cushner S. Sidransky E. (1996a). Differentiation of the glucocerebrosidase gene from pseudogene by long-template PCR: implications for Gaucher disease. Am. J. Hum. Genet. 59, 740–741. 8751878 PMC1914901

[B37] Tayebi N. Stern H. Dymarskaia I. Herman J. Sidransky E. (1996b). 55-base pair deletion in certain patients with Gaucher disease complicates screening for common Gaucher alleles. Am. J. Med. Genet. 66, 316–319. 8985494 10.1002/(SICI)1096-8628(19961218)66:3<316::AID-AJMG15>3.0.CO;2-P

[B38] Tayebi N. Stubblefield B. K. Park J. K. Orvisky E. Walker J. M. LaMarca M. E.. (2003). Reciprocal and nonreciprocal recombination at the glucocerebrosidase gene region: implications for complexity in Gaucher disease. Am. J. Hum. Genet. 72, 519–534. 10.1086/367850 12587096 PMC1180228

[B39] Velayati A. Knight M. A. Stubblefield B. K. Sidransky E. Tayebi N. (2011). Identification of recombinant alleles using quantitative real-time PCR implications for Gaucher disease. J. Mol. Diagn. 13, 401–405. 10.1016/j.jmoldx.2011.02.005 21704274 PMC3123786

[B40] Vithayathil J. Gibney G. Baxevanis A. D. Stubblefield B. K. Sidransky E. Tayebi N. (2009). Glucocerebrosidase mutation H255Q appears to be exclusively in cis with D409H: structural implications. Clin. Genet. 75, 503–504. 10.1111/j.1399-0004.2009.01163.x 19459886 PMC3341623

[B41] Walley A. J. Harris A. (1993). A novel point mutation (D380A) and a rare deletion (1255del55) in the glucocerebrosidase gene causing Gaucher's disease. Hum. Mol. Genet. 2, 1737–1738. 10.1093/hmg/2.10.1737 8268935

[B42] Winfield S. L. Tayebi N. Martin B. M. Ginns E. I. Sidransky E. (1997). Identification of three additional genes contiguous to the glucocerebrosidase locus on chromosome 1q21: implications for Gaucher disease. Genome Res. 7, 1020–1026. 10.1101/gr.7.10.1020 9331372 PMC310674

[B43] Zampieri S. Cattarossi S. Bembi B. Dardis A. (2017). GBA analysis in next-generation era: pitfalls, challenges, possible solutions. J. Mol. Diagn. 19, 733–741. 10.1016/j.jmoldx.2017.05.005 28727984

[B44] Zeng Q. Leach N. T. Zhou Z. Zhu H. Smith J. A. Rosenblum L. S.. (2020). A customized scaffolds approach for the detection and phasing of complex variants by next-generation sequencing. Sci. Rep. 10:15060. 10.1038/s41598-020-71471-3 32929119 PMC7490669

[B45] Zimran A. Horowitz M. (1994). RecTL: a complex allele of the glucocerebrosidase gene associated with a mild clinical course of Gaucher disease. Am. J. Med. Genet. 50, 74–78. 10.1002/ajmg.1320500116 8160756

